# Screening student drinking behaviors: examining AUDIT criterion validity using CIDI-based alcohol use disorder as the ‘gold standard’

**DOI:** 10.3389/fpubh.2024.1328819

**Published:** 2024-04-26

**Authors:** Jens Christoffer Skogen, Mikkel Magnus Thørrisen, Ann Kristin Skrindo Knudsen, Anne Reneflot, Børge Sivertsen

**Affiliations:** ^1^Department of Health Promotion, Norwegian Institute of Public Health, Oslo, Norway; ^2^Centre for Evaluation of Public Health Measures, Norwegian Institute of Public Health, Oslo, Norway; ^3^Center for Alcohol and Drug Research (KORFOR), Stavanger University Hospital, Stavanger, Norway; ^4^Department of Rehabilitation Science and Health Technology, Faculty of Health Sciences, OsloMet – Oslo Metropolitan University, Oslo, Norway; ^5^Department of Public Health, Faculty of Health Sciences, University of Stavanger, Stavanger, Norway; ^6^Department of Disease Burden, Norwegian Institute of Public Health, Oslo, Norway; ^7^Department of Mental Health and Suicide, Norwegian Institute of Public Health, Oslo, Norway; ^8^Department of Research and Innovation, Helse-Fonna HF, Haugesund, Norway

**Keywords:** alcohol use disorder, AUDIT, screening, students, CIDI, drinking, validity

## Abstract

**Introduction:**

High levels of alcohol consumption among college students have been observed across countries. Heavy drinking episodes are particularly prevalent in this population, making early identification of potentially harmful drinking critical from a public health perspective. Short screening instruments such as the Alcohol Use Disorders Identification Test (AUDIT) are serviceable in this regard. However, there is a need for studies investigating the criterion validity of AUDIT in the student population. The aim was to examine the criterion validity of the full AUDIT and AUDIT-C (the first three items directly gauging consumption patterns) in a sample of college and university students using 12-month prevalence of alcohol use disorder derived from an electronic, self-administered version of the World Health Organization (WHO) Composite International Diagnostic Interview, fifth version (CIDI 5.0), which serves as the ‘gold standard’.

**Methods:**

The study population of the current study is derived from the SHoT study (*Students’ Health and Wellbeing Study*), which is a large national survey of students enrolled in higher education in Norway. In a follow-up study of mental disorders among participants of the SHoT2022 study, students were invited to complete a self-administered electronic version of the CIDI. A random sample of 4,642 participants in the nested CIDI-sample was asked to fill out a set of screening instruments, including AUDIT, before starting CIDI. Based on Youden Index maximization, we estimated the sex-specific optimal cut-offs for AUDIT and AUDIT-C in relation to alcohol use disorder, as determined by CIDI.

**Results:**

For the full AUDIT, the optimal cut-offs were 9 for males and 10 for females. The corresponding cut-offs for AUDIT-C were 6 for males and 5 for females. The same optimal cut-offs for both the full AUDIT and AUDIT-C were replicated in bootstrapped analyses with 1,000 runs.

**Conclusion:**

The full AUDIT demonstrated acceptable criterion validity with a balance between sensitivity and specificity. However, for AUDIT-C, caution should be exercised when interpreting screening results among college and university students. In conclusion, the full AUDIT is a reliable screening instrument for college and university students, while further modification may be needed for AUDIT-C in this setting.

## 1 Introduction

Alcohol consumption is a major public health concern, contributing to approximately 5 % of global mortality and disability-adjusted life years (1). According to the Global Burden of Disease-project, alcohol consumption is the leading risk factor for ill-health among those aged 15–49 years old (2). Heavy use of alcohol is associated with various risky behaviors and detrimental outcomes, such as engaging in unprotected sexual activities (3), increased suicide attempts (4), higher rates of violence (5), and an elevated risk of traffic accidents (6). Among those 40 years of age or younger, the disease burden attributed to alcohol consumption is mostly driven by injuries and accidents, while the burden is driven by non-communicable diseases, as well as alcohol use disorder, among those over 40 years (7).

High levels of alcohol consumption among college students have been observed across countries (8–13). Particularly, heavy (binge) drinking episodes are prevalent among college students (14). Addressing such potentially harmful consumption at an early stage is of utmost importance. Young adulthood represents a critical period characterized by substantial life changes and events that influence drinking behaviors. Research has demonstrated a prospective association between heavy drinking during college years and the subsequent development of alcohol dependence in adulthood (15). Furthermore, early adulthood aligns with a pivotal phase of neurocognitive maturation (16), heightening the pertinence of excessive drinking during this period as a risk factor for adverse neurocognitive outcomes.

The Alcohol Use Disorders Identification Test (AUDIT) was developed as a screening instrument for hazardous, harmful and dependent drinking, based on a WHO collaborative project (17). The AUDIT has demonstrated psychometric properties superior to other alcohol screening instruments (18, 19). The AUDIT consists of 10 questions, each scored from 0 to 4, resulting in a potential scoring range of 0 to 40. The AUDIT-C (20), an abbreviated version of the AUDIT (utilizing only the first three items directly gauging consumption patterns), is scored on a scale ranging from 0 to 12.

Initially recommended by the World Health Organization (WHO) (21), the full 10-item AUDIT scale employs specific cut-off values: ≥8 for identifying hazardous drinking, ≥16 for detecting harmful drinking, and ≥ 20 for indicating a probable alcohol dependence. However, some studies have challenged these threshold values (22–24), and some have recommended sex-specific cut-off values, with lower thresholds for females than for males (25, 26). Furthermore, there is inconsistency in the terminology used for risk categories across studies. For instance, some studies employ terms such as “risky drinking,” “alcohol abuse” and “alcohol dependence” instead of the initial WHO terminology. This makes direct comparisons between studies difficult to accomplish.

For the full AUDIT, a threshold of ≥13 has been identified as a suitable balance between sensitivity and specificity for detecting alcohol dependence among Spanish students, with lower cut-offs for moderate and high-risk drinking (≥8 for males, ≥6 for females) (27, 28). In a Nigerian student sample, Adewuya (29) found that a cut-off of ≥5 indicated the presence of alcohol-related problems for both males and females. Among students in the United States, Small et al. (30) suggested a cut-off of ≥8 for detecting alcohol abuse according to DSM-IV criteria, while Hagman (31) conducted a study which indicated that ≥9 (males) and ≥ 8 (females) were indicative of alcohol use disorders according to DSM-5 criteria. Villarosa-Hurlocker et al. (32) proposed ≥12 (males) and ≥ 8 (females) as cut-offs indicating the presence of alcohol use disorders based on DSM-5 criteria.

Regarding the AUDIT-C, Campbell and Maisto (33) recommended thresholds of ≥7 for males and ≥ 5 for females to identify at-risk drinking among students in the United States. Lower cut-offs (≥5 for males and ≥ 4 for females) were suggested for high-risk drinking among Spanish students (28). Hagman (34) examined students in the United States and proposed sex-specific thresholds of ≥5 (males) and ≥ 3 (females) for detecting alcohol use disorders based on DSM-5 criteria.

In sum, studies exploring appropriate cut-off values in college student populations have yielded inconsistent results. Further research on appropriate cut-off values for the AUDIT and the AUDIT-C in college student populations is warranted.

### 1.1 Aims

To examine the criterion validity of the full AUDIT and AUDIT-C in sample of college and university students using 12-month prevalence of alcohol use disorder derived from the electronic, self-administered version of the World Health Organization (WHO) Composite International Diagnostic Interview, fifth version (CIDI 5.0) as the ‘gold standard’. To the best our knowledge, this is the first study to employ this approach to ascertain optimal cut-off values for AUDIT.

## 2 Methods

### 2.1 Setting and participants

The study population of the current study is derived from the SHoT study (*Students’ Health and Wellbeing Study),* which is a large national survey of all students enrolled in higher education in Norway, conducted by the Norwegian Institute of Public Health (NIPH) and the three largest student welfare organizations in Norway. Four main surveys have been completed since its inception in 2010. The current study is based on data from 2022, the most recent wave. The SHoT2022 encompassed a wide range of domains, including mental health and lifestyle factors, and was distributed electronically through a web-based platform. SHoT2022 was conducted between February 8 and April 19, 2022, and invited Norwegian students pursuing higher education, both in Norway and abroad. The students were invited via email and SMS and included both students studying in Norway and Norwegian students studying abroad. All of the invited students were 18 years of age or more, but almost half of the participants were under 23 years of age. In total, 169,572 students fulfilled the inclusion criteria, of whom 59,544 students completed the online questionnaires (after being sent two reminders), yielding a response rate of 35.1%. Details of SHoT have been published elsewhere (35).

Upon consenting to participate in the SHoT2022, students were also asked to indicate if they wished to be invited to a follow-up study of mental disorders, and 26,311 (44%) agreed. This follow-up employed A recently designed electronic, self-administered version of the WHO Composite International Diagnostic Interview, fifth version (CIDI 5.0). To approximate a similar sex distribution as in the base study population, comparatively more males than females were invited to take part in the electronic version of the CIDI study, yielding an invited sample of 16,418 students. Of these 9,911 completed the section assessing alcohol use disorder in the electronic version of the CIDI (response rate = 60.4%). A random sample of 5,076 participants of the nested CIDI-sample were asked to fill out a set of screening instruments before starting the CIDI assessment. A total of 4,642 participants had valid responses on the AUD-section of CIDI, and constitute the study sample in the present study. The electronic version of the CIDI study was conducted between January 24 and February 6 (36). The representativeness of the nested CIDI-sample was investigated in a previous publication by Sivertsen et al. (37). In short, the sociodemographic characteristics among CIDI-participants were largely in correspondence with the overall SHoT2022-study, except for a slight overrepresentation of females (36). The study was approved by the Regional Committee for Medical and Health Research Ethics in Western Norway (no. 2022/326437).

### 2.2 Variables

#### 2.2.1 Sociodemographic information

Sex was determined by the question "What was your sex at birth?", with the options "Male" and "Female".

#### 2.2.2 Alcohol use disorders identification test: AUDIT

As part of the nested CIDI-sample with pre-screening, the participants were asked to complete the Norwegian version of the 10-item alcohol use disorders identification test (AUDIT) before starting CIDI. AUDIT is a screening instrument developed to identify hazardous, harmful and dependent drinking during the past 12 months. It is commonly used worldwide both in research and clinical settings. The included items gauges alcohol consumption and aspects related to alcohol dependence and alcohol-related harm (see Table 1). On item 1–8, the scores can be 0, 1, 2, 3 or 4 and on items 9 and 10 scores can be 0, 2 or 4—with higher scores indicating more alcohol-related problems. Cronbach’s α was 0.81 (CI 95% 0.80–0.82) for the full AUDIT in the present study.

**Table 1:** Overview of AUDIT-items, response options and scoring.

Item #	Response options
1. How often do you have a drink containing alcohol?	**(0)** Never,**(1)** Monthly or less,**(2)** 2–4 times a month,**(3)** 2–3 times a week,**(4)** 4 or more times a week
2. How many standard drinks containing alcohol do you have on a typical day when drinking?	**(0)** 1 or 2,**(1)** 3 or 4,**(2)** 5 or 6,**(3)** 7, 8 or 9 **(4)** 10 or more
3. How often do you have six or more drinks on one occasion?	**(0)** Never,**(1)** Less than monthly,**(2)** Monthly,**(3)** Weekly,**(4)** Daily or almost daily
4. During the past year, how often have you found that you were not able to stop drinking once you had started?	**(0)** Never,**(1)** Less than monthly,**(2)** Monthly,**(3)** Weekly,**(4)** Daily or almost daily
5. During the past year, how often have you failed to do what was normally expected of you because of drinking?	**(0)** Never,**(1)** Less than monthly,**(2)** Monthly,**(3)** Weekly,**(4)** Daily or almost daily
6. During the past year, how often have you needed a drink in the morning to get yourself going after a heavy drinking session?	**(0)** Never,**(1)** Less than monthly,**(2)** Monthly,**(3)** Weekly,**(4)** Daily or almost daily
7. During the past year, how often have you had a feeling of guilt or remorse after drinking?	**(0)** Never,**(1)** Less than monthly,**(2)** Monthly,**(3)** Weekly,**(4)** Daily or almost daily
8. During the past year, how often have you been unable to remember what happened the night before because you had been drinking?	**(0)** Never,**(1)** Less than monthly,**(2)** Monthly,**(3)** Weekly,**(4)** Daily or almost daily
9. Have you or someone else been injured as a result of your drinking?	**(0)** No,**(2)** Yes, but not in the past year,**(4)** Yes, during the past year
10. Has a relative or friend, doctor or other health worker been concerned about your drinking or suggested you cut down?	**(0)** No,**(2)** Yes, but not in the past year,**(4)** Yes, during the past year

Although previous studies have found support for different factor structures of AUDIT, the most common way to use AUDIT is as a unidimensional measure, and this approach is also supported by a study in a Norwegian context (38). As the first three items (AUDIT-C) is often used as a brief screening tool for potential unhealthy alcohol use, we investigate the criterion validity of both the full AUDIT and AUDIT-C in the present study.

#### 2.2.3 Alcohol use disorder: the self-administered electronic version of CIDI

A recently designed electronic, self-administered version of the WHO Composite International Diagnostic Interview, fifth version (CIDI 5.0), developed for the WHO World Mental Health (WMH) Surveys was used for the data-collection (39). This self-administered electronic version of the CIDI was developed by The World Mental Health Survey Initiative, at Harvard University. The electronic version of the CIDI was implemented in Blaise 5.4, a software tool designed to collect survey data. Blaise is used by several national statistics agencies in Europe, and Statistics Norway administered the Norwegian translation of the electronic version of the CIDI used in the present study and conducted the data collection.

CIDI 5.0 is a standardized interview assessing 30-days, 12 months and lifetime prevalence for several mental and substance use disorders according to diagnostic criteria in the Diagnostic and Statistical Manual of Mental Disorders 5th edition (DSM-5) (40). CIDI 5.0 has good agreement with other prevailing diagnostic instruments such as the Structured Clinical Interview for DSM-IV (SCID) (41) and Schedules for Clinical Assessment in Neuropsychiatry (SCAN) (42). The Norwegian version of the electronic CIDI is based on the official Norwegian translation of CIDI. 5.0, as described in a previous study protocol publication (43).

For the purposes of the present study, one outcome measure was employed, namely 12-month prevalence of alcohol use disorder. Operationalization of the diagnosis was based on algorithms developed for CIDI 5.0 by WMH. Valid responses were not required to progress in the electronic version of the CIDI survey, and the final sample constitutes participants who completed the alcohol-section of the CIDI.

### 2.3 Statistical analyses

**Figure 1:**
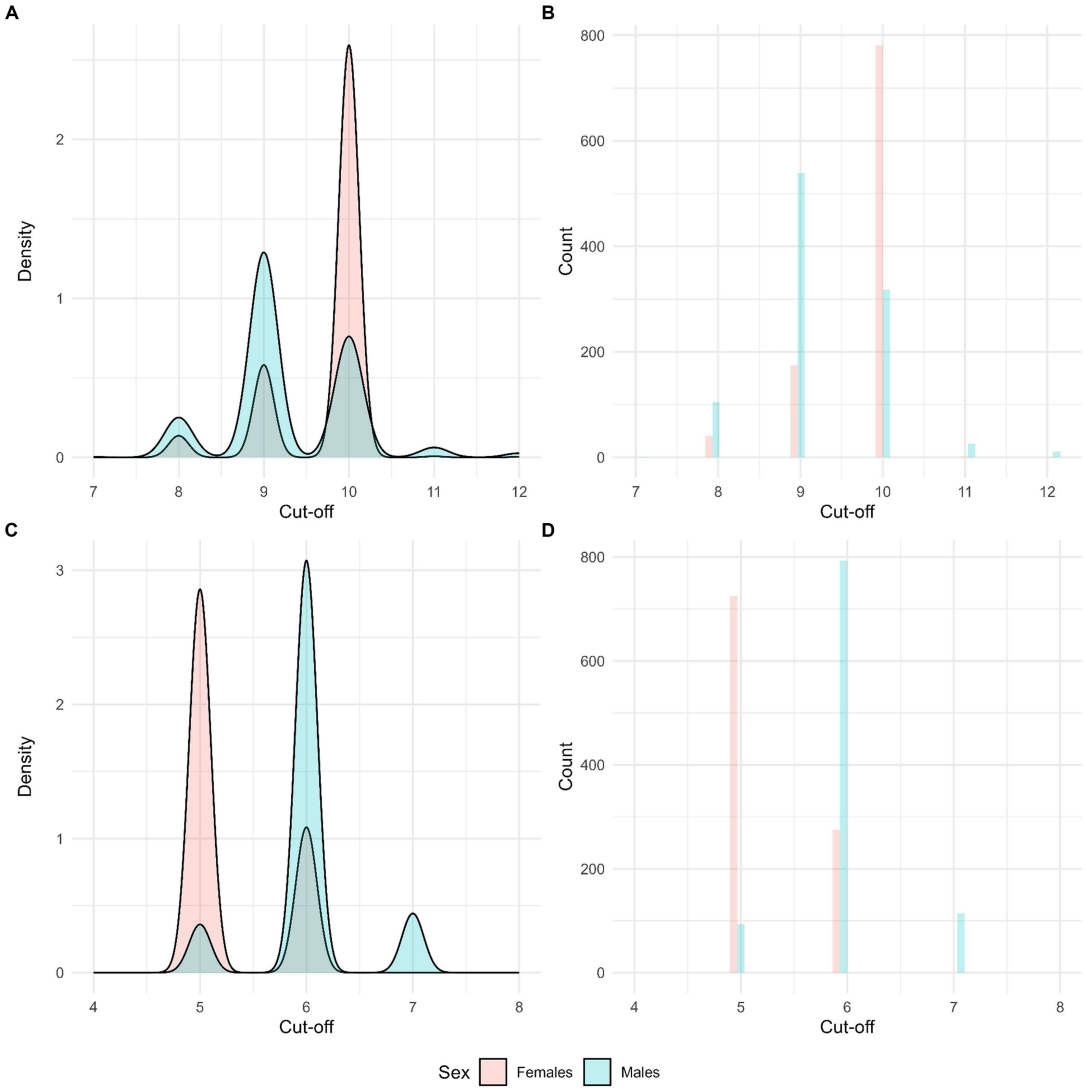
Results from bootstrapped analysis. Count and density plots for optimal cut-offs. Full AUDIT **(A,B)** and AUDIT-C **(C,D)**. 1,000 boot runs. Stratified by sex.

**Figure 2:**
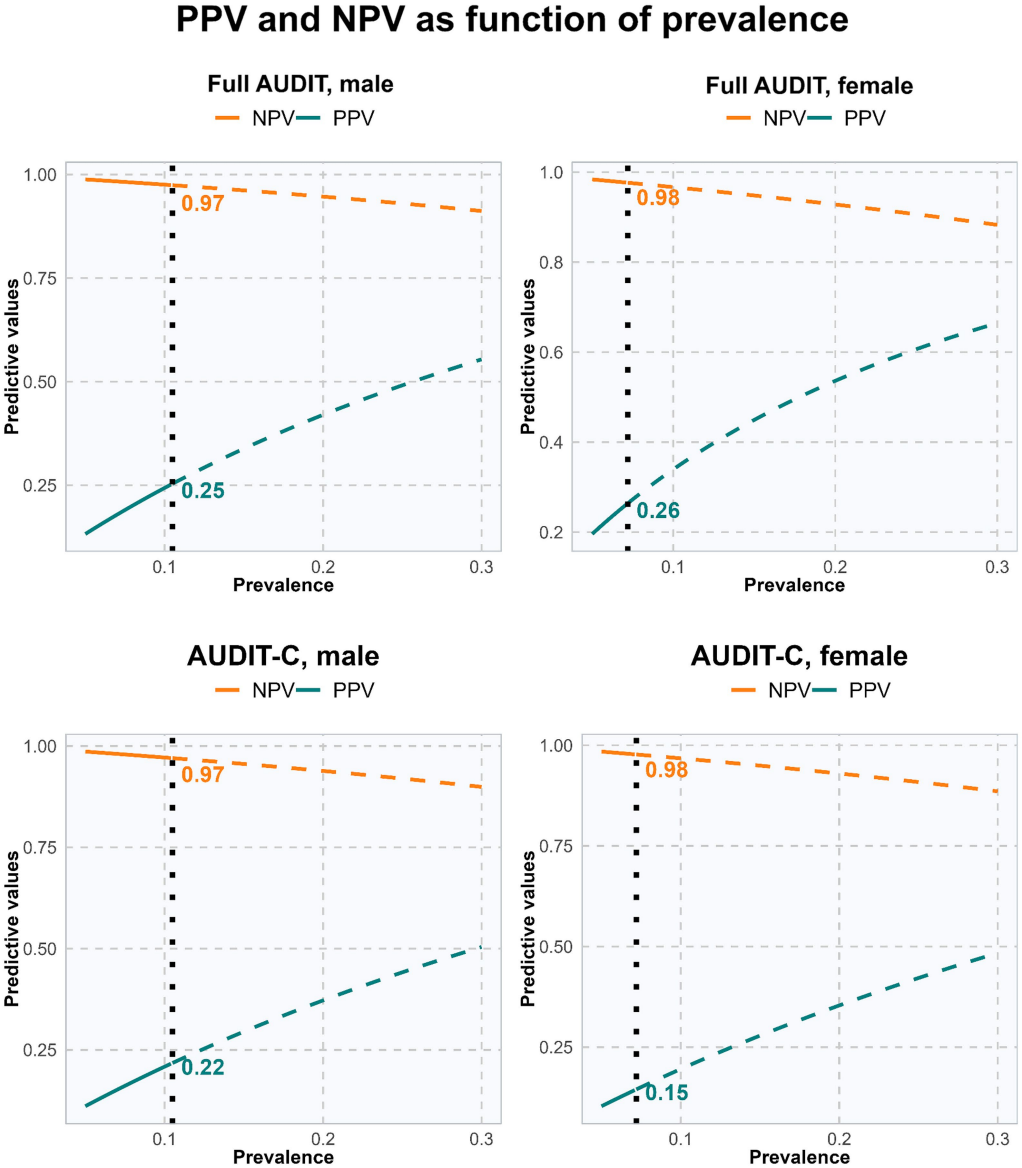
Positive (PPV) and negative (NPV) predictive values as function of prevalence. PPV and NPV highlighted at observed 12-month prevalence of alcohol use disorder using vertical dotted line. Full AUDIT and AUDIT-C. Stratified by sex.

In the present study we first present summary statistics of the AUDIT-scores and prevalence of alcohol use disorder stratified by sex (Table 2). To assess the factor structure of the full AUDIT, we performed an exploratory graph analysis and confirmatory factor analysis (CFA). CFA was conducted using an estimator suitable for ordinal scaled variables (diagonally weighted least squares; DWLS), and RMSEA, CFI and TLI was used to determine model fit. Next, the sex-specific optimal cut-offs for AUDIT and AUDIT-C in relation to alcohol use disorder (as determined by the CIDI) was estimated based on Youden Index maximization (Table 3). Youden Index is a commonly used metric for binary classification in validation studies and aims to strike a balance between sensitivity and specificity. The formula for the Youden Index is *(‘sensitivity’ + ‘specificity’) -1*, and the index ranges from 0 to 1. A higher value indicates better discriminative ability, where 0 is no discrimination and 1 is perfect discrimination. Although, rule of thumbs always must be considered in conjunction with other aspects, a score below 0.5 on the Youden Index indicates that the test in question may not be useful classification, while a score over 0.5 may be interpreted as a useful test. To test the stability of the identified optimal cut-offs, a bootstrapped sex-specific analysis was conducted with 1,000 runs for the full AUDIT and AUDIT-C (Figure 1; Supplementary Table 1). In addition to the Youden Index, the overall accuracy, sensitivity, specificity, and area under the curve (AUC) are presented in Table 3. The positive (PPV) and negative (NPV) predictive values for the optimal cut-offs are presented in Figure 2 as a function of prevalence. As a sensitivity test, we also investigated alternative sex-specific cut-offs for the full AUDIT and AUDIT-C with a tolerance set to +/− 0.20 on the Youden Index (Supplementary Table 2). All analyses were done using R (44), and R Studio [Posit team (36)] and the following packages ‘EGAnet’, ‘Lavaan’, ‘gtsummary’ and ‘cutpointr’ (45–48). Missing was handled by case-wise deletion.

**Table 2:** Summary statistics across sex.

Variables	Males, *N* = 1384^1^	Females, *N* = 3258^1^	*p*-value^2^
AUDIT Total score	7.11 (4.96)/7.00	6.17 (4.49)/6.00	<0.001
AUDIT-C scores	4.60 (2.52)/5.00	3.90 (2.18)/4.00	<0.001
DSM-5 12 Month Alcohol Use Disorder	145 (10.5%)	236 (7.2%)	<0.001

^1^Mean (SD)/Median; n (%). ^2^Wilcoxon rank sum test; Pearson’s Chi-squared test.

**Table 3:** Optimal cut-offs for AUDIT and AUDIT-C stratified by sex.

Sex		Cut-offs	Youden index	ACC	Sensitivity	Specificity	AUC	Prevalence
Full AUDIT								
Male	←	9	0.55	0.72	0.84	0.71	0.85	0.105
Female	←	10	0.58	0.84	0.74	0.84	0.86	0.072
AUDIT-C								
Male	←	6	0.48	0.67	0.83	0.65	0.80	0.105
Female	←	5	0.44	0.64	0.81	0.63	0.78	0.072

ACC, Accuracy; AUC, Area under the curve.

## 3 Results

Summary statistics are presented in Table 2. Males reported higher scores on AUDIT and AUDIT-C and the prevalence of 12-month alcohol use disorder was higher among males as well. The exploratory graph analysis indicated a unidimensional structure of the full AUDIT, and this finding was also replicated in bootstrapped results with 500 runs (see Supplementary Figure 1). The CFA yielded support for a 1-factor structure (RMSEA: 0.054 (90%CI 0.049–0.058), CFI: 0.989, TLI: 0.985, SRMR: 0.047), with overall good model fit, albeit with correlated error terms between consumption item 1 and consumption items 2 and 3. For the full AUDIT, the optimal cut-offs were 9 for males and 10 for females, while the corresponding scores were 6 and 5 on AUDIT-C (see Table 3; Figure 1). The same optimal cut-offs for both the full AUDIT and AUDIT-C were replicated in bootstrapped analyses with 1,000 runs (see Supplementary Table 1).

### 3.1 Full AUDIT

For the full AUDIT, there was an acceptable balance between sensitivity and specificity, and the Youden Index was acceptable (>0.50) for both males and females. The PPV and NPV for males were 0.25 and 0.97, while the corresponding numbers were 0.26 and 0.98 for females (see Figure 2). Using the optimal cut-offs would identify 34.9% of the males and 19.7% of the females in the sample as case-positive according to the full AUDIT, compared to 10.5 and 7.2%, respectively, according to the CIDI. Additional sensitivity analyses indicated that a better balance between sensitivity and specificity could be obtained by changing the cut-off to 10 for males and to 9 for females, but at the cost of a slightly lower Youden Index score (see Supplementary Table 2). This would augment the PPV to 0.29 and 0.30 for males and females, respectively. See Supplementary Table 2 for other potential cut-offs for the full AUDIT.

### 3.2 AUDIT-C

For AUDIT-C, there was an imbalance between sensitivity and specificity with substantially higher values on sensitivity than specificity for both males and females. This is also reflected in the Youden Index for both sexes, which is less than acceptable (≤0.50). The PPV and NPV for males were 0.22 and 0.97, while the corresponding numbers were 0.15 and 0.98 for females (see Figure 2). Using the optimal cut-offs would identify 40.2% of the males and 40.0% of the females as case-positive according to AUDIT-C, compared to 10.5 and 7.2%, respectively according to the CIDI. See Supplementary Table 2 for other potential cut-offs for AUDIT-C.

## 4 Discussion

In the present study we examined the criterion validity of the full AUDIT and AUDIT-C in a sample of college and university students using 12-month prevalence of alcohol use disorder identified through the diagnostic instrument CIDI 5.0 as the ‘gold standard’. Using the maximization of the Youden Index as the preferred approach, the recommended cut-offs for the full AUDIT were 9 and 10 for males and females, respectively. For AUDIT-C, the recommended cut-offs were 6 for males and 5 for females. Overall, our findings indicate acceptable criterion validity of the full AUDIT based on the maximization of the Youden Index, while the AUDIT-C had lower-than-acceptable criterion validity in our sample. This is not to say that AUDIT-C cannot be used as a screening instrument in the studied population, but extra caution should be taken into consideration when interpreting the results using AUDIT-C in a college or university setting. Across the two versions of AUDIT examined, the negative predictive value was high for both sexes, while the positive predictive value was low, especially for AUDIT-C among females. As a case in point, given current findings, screening 1,000 male students with the full AUDIT would identify 349 as case-positive, but only approximately 88 out of them would be correctly classified according to CIDI 5.0. Conversely, 651 of the male students would be designated as non-cases, and this would be correct for approximately 634 of them.

For males, and in alignment with Hagman’s (2016) study on students in the United States, our findings suggest that a cut-off score of ≥9 on the full AUDIT is indicative of alcohol use disorders, as defined by the DSM-5 criteria. However, our recommended threshold of ≥6 for males on the AUDIT-C is slightly lower than the at-risk drinking threshold of ≥7 identified among students in the United States (33), and higher than the thresholds identified for high-risk drinking among Spanish students (≥5) (28) and alcohol use disorders among American students (≥5) (34). For females, our study on Norwegian students generally indicated higher cut-off values than those found in previous examinations of student populations. Specifically, we identified a cut-off score of ≥10 on the full AUDIT, and ≥ 5 on the AUDIT-C for females. In contrast, previous studies have suggested cut-off scores on the full AUDIT for females ranging from ≥6 to ≥8 (27, 28, 31, 32), while the corresponding range on the AUDIT-C has been identified between ≥3 and ≥ 5 (28, 33, 34).

In general, various factors may contribute to differences in reported cut-off points across studies. These factors encompass heterogeneity in study populations and baseline prevalence rates, variations in outcomes of interest and their operationalization, cultural and ethnic disparities, temporal shifts in the nature of the outcomes of interest, and differences in analytical methodologies and research objectives. In relation to AUDIT, it must be noted that the lack of standardized terminology for risk categories constitutes a barrier to understanding similarities and differences in cut-off values across studies (23). For instance, while the initial WHO recommendations were linked to “hazardous,” “harmful” and “dependence likely” drinking (17, 21), other concepts have frequently been used across studies (e.g., “at-risk drinking,” “moderate-risk drinking,” “high-risk drinking,” “alcohol-related problems,” “alcohol use disorders,” “alcohol abuse” and “alcohol dependence”). To the best of our knowledge, no previous study has compared the criterion validity of AUDIT using an electronic version of a self-administered standardized diagnostic interview protocol, either in a college/university setting or in the general population – shedding new light on the case-finding ability of AUDIT.

### 4.1 Implications and future research

For both the full AUDIT and AUDIT-C, it was not possible to strike a very good balance between sensitivity and specificity while holding both at a high level. This was especially true for AUDIT-C which only gauges consumption patterns. Depending on the purpose and context, different cut-offs might be more or less advantageous. In our sample for instance lowering the cut-off for the full AUDIT would markedly increase the sensitivity and decrease the specificity considerably—while the PPV would increase substantially at only a slight decrease in NPV (see Supplementary Figure 2 for an example). Although the effect on PPV and NPV is contingent on the prevalence, in some scenarios it might be desirable to sacrifice specificity for sensitivity or the other way around. If the goal is to capture as many true cases as possible during initial screening this approach may be serviceable. The estimated 12-month prevalence of alcohol use disorder was relatively high in the present population among both men and women, and the prevalence is likely to be lower in a general population of adults and higher in for instance a clinical setting. However, the reported prevalence for males and females in the present study is comparable to estimates from the Dutch NEMESIS-study of 12-month alcohol use disorder among university and applied science students; 9.7% among males and 5.3% among females (49). It is also close to age-specific estimates from the same study where the prevalence is reported to be 16.3 and 14.3% among males aged 18–24 and 25–34 years old, and 8.5 and 5.2% among females in the same two age groups but from a general population. The expected or estimated prevalence is also important to consider in conjunction with the purpose of using AUDIT when deciding on a cut-off. Based on our findings, we would generally recommend using the optimal cut-offs presented above for analytical epidemiological studies of college or university students, while other cut-offs can be considered depending on the context and purpose in question. As a brief screening in terms of considering secondary or tertiary prevention measures among college or university students, the optimal cut-offs may be best used as an initial assessment of whether for instance brief alcohol interventions are needed, but further investigation is necessary through follow-up questions or brief interviews if the aim is to identify those with a true alcohol use disorder. As such, AUDIT can be considered suitable as the first part of a multi-phased screening in a practical or clinical setting for college and university students. It is also important to note that alcohol use disorder was used in the present study as the criterion. As such, the aim of the present study was not investigating the optimal cut-off for ‘unhealthy’ or ‘risky’ drinking. As mentioned in the introduction, several different terms are in use to describe alcohol use patterns which is likely to be unhealthy in the short-term, long-term or both. However, the heterogeneity in terminologies and the varied emphasis on different facets of alcohol consumption, such as ‘binge drinking,’ pose challenges for meaningful comparisons in terms of validity. Regardless, establishing optimal cut-offs for AUDIT in respect to consumption patterns are important in their own right, and also due to their potential role as potent risk factors for the onset and progression of alcohol use disorder. Future research should investigate validity of AUDIT for alcohol use disorder in conjunction with other outcomes, such as alcohol-related accidents or alcohol-related harm.

Although the AUDIT is a well-established screening instrument, and the present results indicate that it is useful as a screening instrument for students in relation to alcohol use disorder, more research is needed. A somewhat surprising finding in the present study was that the optimal cut-off for the full AUDIT was higher for females compared to males, while the opposite was true for AUDIT-C. Although the latter is expected based on previous studies, a higher cut-off for females on the full AUDIT warrants further investigation. It may be due to females needing lower levels of alcohol consumption before they experience other issues related to their alcohol use (which are captured by items 4–10 in the AUDIT). In other words, it may be that the consumption items are more sensitive indicators of alcohol use disorders in females compared to males. Future research should also consider investigating whether modifying AUDIT by replacing or adding items could enhance the criterion validity vis-à-vis alcohol use disorder for screening in a college and university setting. For instance, could the inclusion of items directly related to everyday student life be considered, such as missing class or lecture due to being hung over. Other items gauging whether one consumes alcohol alone and on which days (i.e., Mondays versus Fridays) one consumes alcohol may be helpful in increasing the criterion validity. Also, related to the consumption items, increasing the number of response options may help to better discriminate between true cases and non-cases. Future research should also consider other clinically relevant alcohol-related outcomes in relation to the validity of AUDIT in a college or university setting.

### 4.2 Strengths and limitations

The present study holds several strengths. First, the data collection is recent and covers more than 5,000 participants. Thus, we were able to determine optimal sex-specific cut-offs. Second, as the first study globally, we were also able to employ a ‘gold standard’ by leveraging data gained from a newly developed electronic self-administered version of the CIDI 5.0. Although the self-administered electronic version is yet to be validated against face-to-face or telephone interviews, we believe the inherent rigorousness and standardization of the CIDI is maintained. Also, it is possible that self-administered electronic versions are especially suitable when assessing stigma-prone disorders, such as alcohol use disorder. The present study also has some limitations that must be considered. First, the response rate of both the SHoT2022 and the nested CIDI-sample was modest. This could potentially bias our results and be a threat to generalizability. A thorough analysis of non-participation would help to shed light on this potential challenge. Unfortunately, we have no information about non-participants in SHoT2022 (50), and only very limited information about non-participants in the nested CIDI-sample (37). Second, in terms of generalizability, alcohol consumption in Norway is traditionally characterized by heavy episodic drinking (‘binge drinking’), more integrated into social activities such as parties and a higher tolerance to public intoxication compared to ‘dry’ cultures characterized by moderation and drinking as part of meals (51). Different drinking habits and sociocultural differences are likely to impact relationships between AUDIT-scores and diagnostic outcomes (52). This means that our findings do not necessarily translate into other settings with a different alcohol culture, especially when considering AUDIT-C. However, college and university students are in general characterized by higher levels of alcohol consumption compared to other segments of the adult population (53). Third, and related to the alcohol consumption patterns among students—many students consume a lot of alcohol for limited periods (e.g., during the introductory week or at the start of the semester) in their student life. Most do, however, not necessarily experience any other problems related to or adverse consequences of alcohol given the relatively restricted intermittent extent of their higher-level alcohol consumption. In a college or university setting, this aspect may pose challenges in accurately distinguishing individuals with alcohol use disorder from those without, particularly when employing brief screening instruments. Fourth, in the present study, we used alcohol use disorder during the past 12-months as the criterion. Admittedly, other clinical aspects related to alcohol use might also be relevant when considering the usefulness of AUDIT, such as alcohol abuse, alcohol dependence, and alcohol-related harm in general. Using another, but still clinically relevant criterion, would probably lead to other results in terms of the validity of the full AUDIT and AUDIT-C. Fifth, alcohol use disorder was not graded in the present study, and we did not sub-classify according to severity level (i.e., ‘mild’, ‘moderate’ and ‘severe’). This would also impact the estimated criterion validity of AUDIT.

## 5 Conclusion

The present study examined the criterion validity of the full AUDIT and AUDIT-C among college and university students in relation to alcohol use disorder. Optimal sex-specific cut-off scores for both versions of AUDIT were presented and discussed. The full AUDIT demonstrated acceptable criterion validity with a balance between sensitivity and specificity. However, for AUDIT-C, caution should be exercised when interpreting results from screening among college and university students. Different cut-off scores may be more advantageous depending on the purpose in question and expected prevalence of alcohol use disorder. Future research should explore modifications to enhance criterion validity, such as adding items related to student life and increasing response options. In summary, the full AUDIT is a reliable screening instrument for college and university students, while further modification may be needed for AUDIT-C in this setting.

## Data availability statement

The datasets presented in this article are not readily available because Norwegian data protection regulations and GDPR impose restrictions on sharing of individual participant data. However, researchers may gain access to survey participant data by contacting the publication committee (borge.sivertsen@fhi.no). Approval from the Norwegian Regional Committee for Medical and Health Research Ethics (https://helseforskning.etikkom.no) is a pre-requirement for access to the data. The dataset is administrated by the NIPH, and guidelines for access to data are found at: https://www.fhi.no/en/more/access-to-data. Analytic codes for the analyses are available upon reasonable request to the corresponding author. Requests to access the datasets should be directed to BS, borge.sivertsen@fhi.no.

## Ethics statement

The studies involving humans were approved by the Regional Committee for Medical and Health Research Ethics in Western Norway (no. 2022/326437). The studies were conducted in accordance with the local legislation and institutional requirements. The participants provided their written informed consent to participate in this study.

## Author contributions

JS: Conceptualization, Formal analysis, Methodology, Validation, Visualization, Writing – original draft, Writing – review & editing. MT: Methodology, Writing – original draft, Writing – review & editing. AK: Conceptualization, Methodology, Writing – review & editing. AR: Conceptualization, Methodology, Project administration, Writing – review & editing. BS: Conceptualization, Investigation, Methodology, Project administration, Writing – review & editing.
