# Supplementary material for: Screening student drinking behaviors: examining AUDIT criterion validity using CIDI-based alcohol use disorder as the ‘gold standard’
Source: Front Public Health. 2024 Apr 26;12:1328819. doi: 10.3389/fpubh.2024.1328819 (PMC11082383; doi:10.3389/fpubh.2024.1328819)

Supplementary Material

# Supplementary Figures and Tables

**Supplementary Figure 1.** Results from exploratory graph analysis (EGA). Dimensionality (A) and item stability across 500 boot runs (B). Full AUDIT.


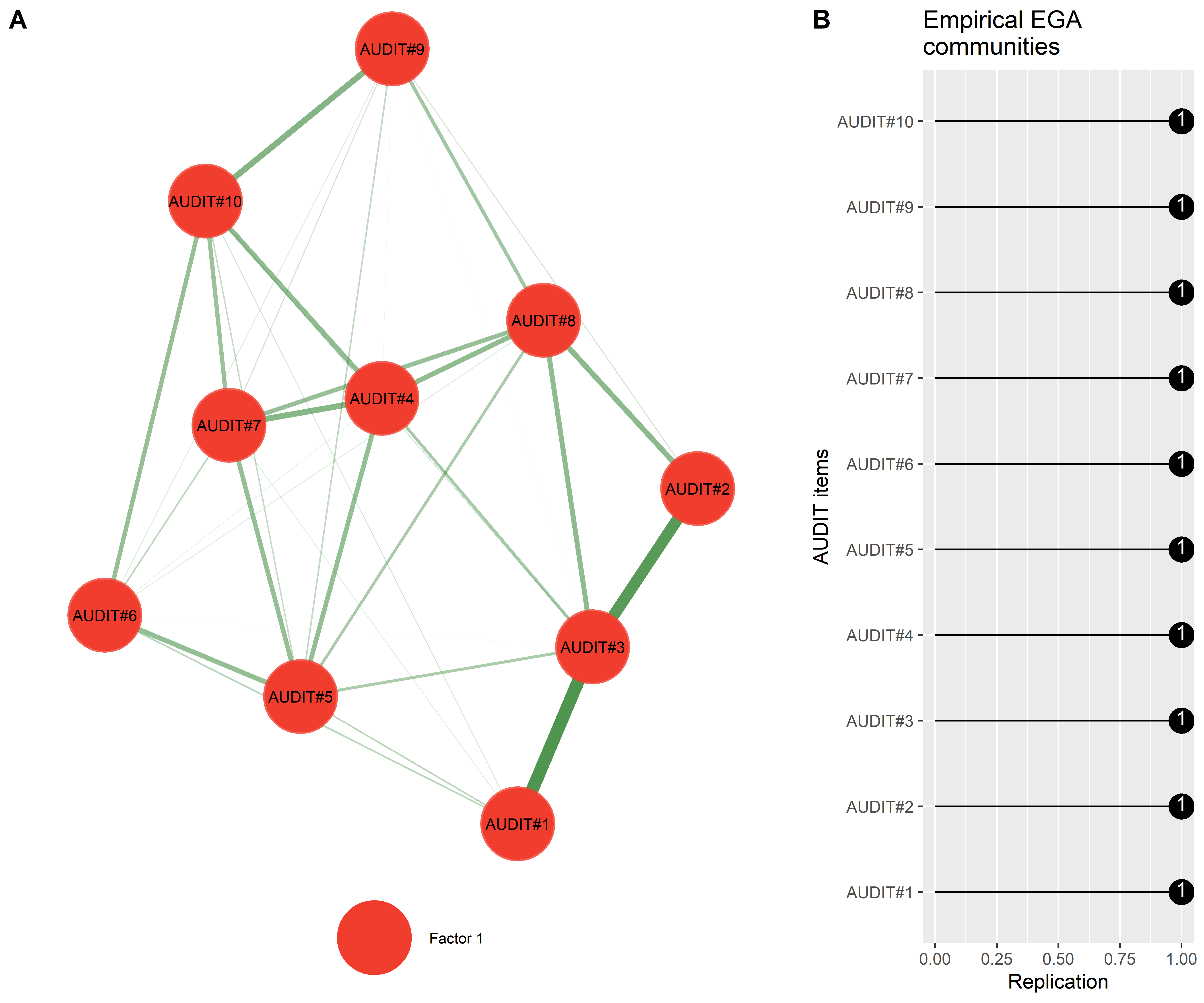


*Results from exploratory graph analysis*

**Supplementary Table 1:** Optimal cut-offs for AUDIT and AUDIT-C stratified by sex. Bootstrapped results. 1,000 boot runs.

| Sex |  | Cut-offs | Youden Index | ACC | Sensitivity | Specificity | AUC | Prevalence |
| --- | --- | --- | --- | --- | --- | --- | --- | --- |
| Full AUDIT |  |  |  |  |  |  |  |  |
| Male | >= | 9 | 0.55 | 0.72 | 0.84 | 0.71 | 0.85 | 0.105 |
| Female | >= | 10 | 0.58 | 0.84 | 0.74 | 0.84 | 0.86 | 0.072 |
| AUDIT-C |  |  |  |  |  |  |  |  |
| Male | >= | 6 | 0.48 | 0.67 | 0.83 | 0.65 | 0.80 | 0.105 |
| Female | >= | 5 | 0.44 | 0.64 | 0.81 | 0.63 | 0.78 | 0.072 |

**Supplementary Table 2:** Alternative cut-offs stratified by sex. Tolerance set to +/- 0.2 on Youden Index-metric.

| Sex | Cut-offs | ACC | Sensitivity | Specificity | Youden Index |
| --- | --- | --- | --- | --- | --- |
| AUDIT |  |  |  |  |  |
| Male | 13 | 0.87 | 0.50 | 0.92 | 0.41 |
| Male | 12 | 0.85 | 0.61 | 0.87 | 0.49 |
| Male | 11 | 0.81 | 0.69 | 0.82 | 0.51 |
| Male | 10 | 0.77 | 0.77 | 0.78 | 0.54 |
| **Male** | **9** | **0.72** | **0.84** | **0.71** | **0.55** |
| Male | 8 | 0.66 | 0.90 | 0.63 | 0.53 |
| Male | 7 | 0.59 | 0.93 | 0.55 | 0.48 |
| Male | 6 | 0.51 | 0.95 | 0.46 | 0.41 |
| Female | 14 | 0.92 | 0.42 | 0.96 | 0.39 |
| Female | 13 | 0.92 | 0.52 | 0.95 | 0.46 |
| Female | 12 | 0.90 | 0.58 | 0.93 | 0.50 |
| Female | 11 | 0.87 | 0.64 | 0.89 | 0.53 |
| **Female** | **10** | **0.84** | **0.74** | **0.84** | **0.58** |
| Female | 9 | 0.78 | 0.79 | 0.78 | 0.57 |
| Female | 8 | 0.71 | 0.85 | 0.70 | 0.55 |
| Female | 7 | 0.64 | 0.89 | 0.62 | 0.51 |
| Female | 6 | 0.56 | 0.92 | 0.53 | 0.45 |
| AUDIT-C |  |  |  |  |  |
| Male | 8 | 0.86 | 0.40 | 0.92 | 0.32 |
| Male | 7 | 0.77 | 0.66 | 0.78 | 0.44 |
| **Male** | **6** | **0.67** | **0.83** | **0.65** | **0.48** |
| Male | 5 | 0.56 | 0.92 | 0.52 | 0.44 |
| Male | 4 | 0.41 | 0.94 | 0.35 | 0.29 |
| Female | 7 | 0.87 | 0.41 | 0.91 | 0.31 |
| Female | 6 | 0.76 | 0.65 | 0.77 | 0.42 |
| **Female** | **5** | **0.64** | **0.81** | **0.63** | **0.44** |
| Female | 4 | 0.46 | 0.92 | 0.42 | 0.33 |

Bold indicates the optimal cut-off based on maximization of Youden Index.

**Supplementary Figure 2:** Positive (PPV) and negative (NPV) predictive values as function of prevalence. Using score of 6 as alternative cut-off to maximize sensitivity. PPV and NPV highlighted at observed 12-month prevalence of alcohol use disorder using vertical dotted lines. Full AUDIT. Stratified by sex.


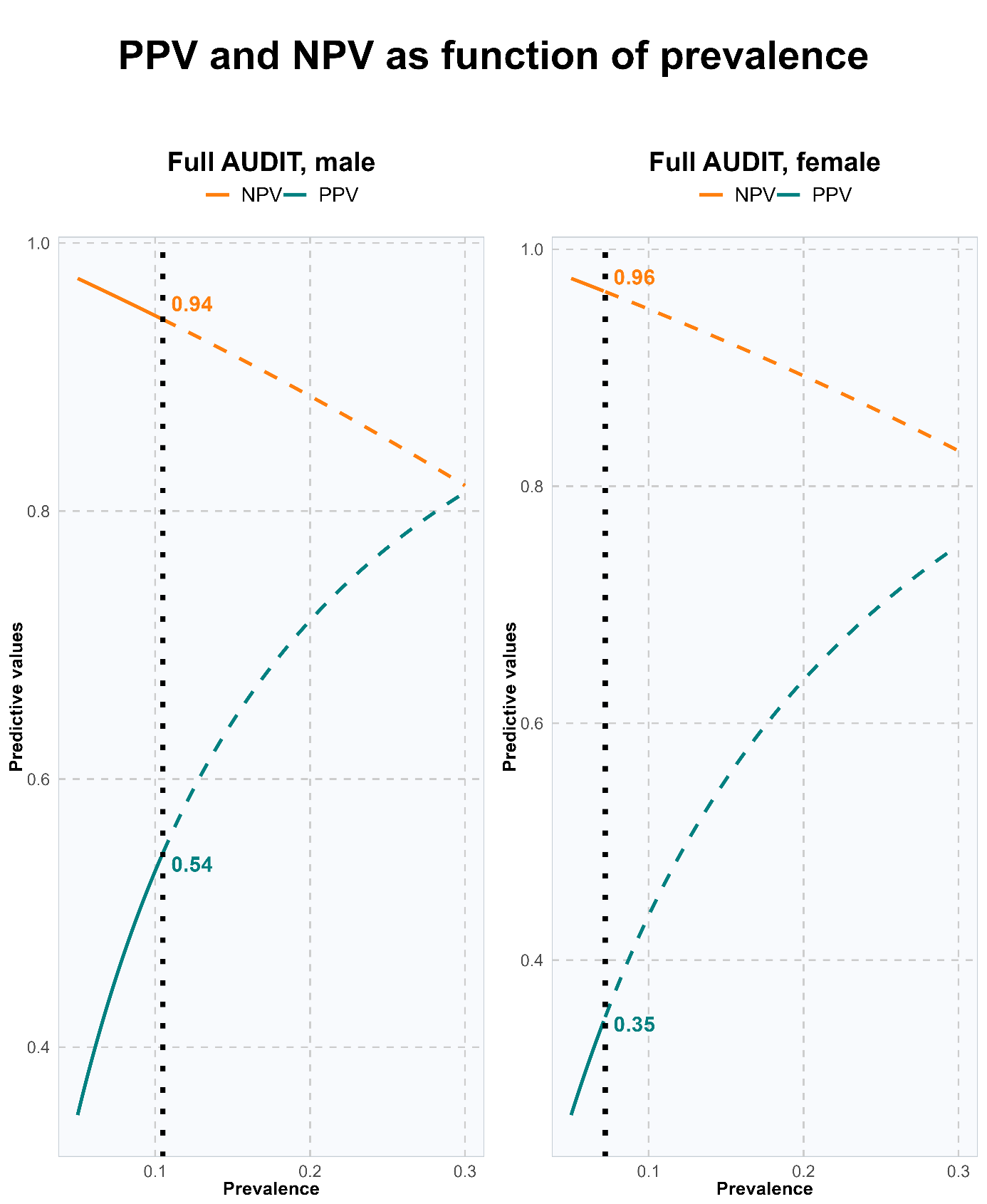

Supplement: Supplementary file 1 [file Data_Sheet_1.docx]
